# Remote ballistic fractures in a gelatine model - aetiology and surgical implications

**DOI:** 10.1186/1749-799X-8-15

**Published:** 2013-05-30

**Authors:** David C Kieser, Debra J Carr, Sandra CJ Leclair, Ian Horsfall, Jean-Claude Theis, Mike V Swain, Jules A Kieser

**Affiliations:** 1 Orthopaedic Surgery, Surgical Sciences, Health Sciences, Dunedin School of Medicine, University of Otago, Dunedin 9054, New Zealand; 2 New Zealand Defence Force, Wellington 5045, New Zealand; 3 Impact and Armour Group, Department of Engineering and Applied Science, Cranfield Defence and Security, Defence Academy of the United Kingdom, Shrivenham, Wiltshire, SN6 8LA, UK; 4 University of Angers, Angers, 49100, France; 5 Sir John Walsh Research Institute, University of Otago, Dunedin, 9054, New Zealand

## Abstract

**Background:**

Remote ballistic femoral fractures are rare fractures reported in the literature but still debated as to their existence and, indeed, their treatment. This study aimed to prove their existence, understand how they occur and determine which ammunition provides the greatest threat. In addition, fracture patterns, soft tissue disruption and contamination were assessed to aid in treatment planning.

**Method:**

We filmed 42 deer femora embedded in ballistic gelatine and shot with four different military (5.56 × 45 mm, 7.62 × 39 mm) and civilian (9 × 19 mm, .44 in.) bullets, at varying distances off the bone (0–10 cm).

**Results:**

Two remote ballistic fractures occurred, both with .44 in. hollow-point bullets shot 3 cm off the bone. These fractures occurred when the leading edge of the expanding temporary cavity impacted the femur's supracondylar region, producing a wedge-shaped fracture with an undisplaced limb, deceivingly giving the appearance of a spiral fracture. No communication was seen between the fracture and permanent cavity, despite the temporary cavity encasing the fracture and stripping periosteum from its base.

**Conclusion:**

These fractures occur with civilian ammunition, but cannot prove their existence with military rounds. They result from the expanding temporary cavity affecting the weakest part of the bone, creating a potentially contaminated wedge-shaped fracture, important for surgeons considering operative intervention.

## Introduction

Gunshot injuries are common not only during armed conflict, but also within the civilian setting [1-5]. Most of these injuries affect the limbs [6-9], with nearly half resulting in a fracture [10].

One of the most intriguing and unexplained fractures is the remote spiral femur fracture, with the bone breaking at a location remote to the bullet's path [11]. Interestingly, most victims describe walking or running a few paces after sustaining the gunshot injury, prior to collapsing [11,12]. Critics deny the existence of this fracture, claiming that the fracture occurs whilst falling and not directly related to the gunshot injury.

With armed conflict escalating globally, it has become increasingly difficult for both military and civilian surgeons to ignore this rare fracture. However, to date, no further research has been presented to conclusively prove its existence, let alone suggest the optimal management for this injury. In order to assess the capability of the four most common military (5.56 × 45 mm, 7.62 × 39 mm) and civilian (9 × 19 mm, .44 in.) bullets in causing this fracture, we developed a gelatine model and analyzed how these fractures occur, described their fracture patterns, extent of periosteal disruption and potential contamination, to aid in surgical decision making.

## Method

The research performed during this experiment was conducted under the ethical approval of the University of Otago Animal Ethics Committee (No. 68/11) and performed according to the principles of ethical research practice, as described in the eighth edition of the *Guide for the Care and Use of Laboratory Animals* published by the National Academy of Sciences, The National Academies Press, Washington, D.C.

Forty-two adult female red deer (*Cervus elaphus*) rear femora, obtained on the day of slaughter, were debrided of soft tissue, leaving the periosteum intact, and kept moist with saline-soaked gauze (0.9% NaCl) refrigerated at 4°C. All bones were of the same size, with no macroscopically visible differences. Thirty-eight of these bones were embedded to a depth of 8 cm in 18 cm (depth) × 18 cm (breadth) × 30 cm (length) rectangular containers of 20% 250B ballistic gelatine (Weishardt International, Graulhet, France). Their anterior cortex faced the surface of the gel, and their long axis paralleled that of the gel. The remaining four bones were embedded in the anatomical position of a thigh-shaped mould of the same dimensions and gelatine consistency as the rectangular moulds. The samples were left to solidify overnight at room temperature (8°C), before being positioned 10 m from a number 3 Enfield pressure housing fitted with appropriate barrels. The bone's anterior cortex was positioned to face the barrel, and the bullet's path was aimed with a barrel-mounted laser to either hit the bone or to pass with varying distances (up to 10 cm) medial to the medial cortex of the bone.

A slow-motion camera (Phantom V12, Vision Research, Inc., Wayne, NJ, USA; 40,000 frames per second) was positioned on the lateral side of each block and a 45° mirror was positioned above the sample, giving synchronized images in the sagittal and axial planes.

Fracture displacement was defined as the movement of the fracture site away from its pre-impact position, whereas fracture end separation was defined as the maximal distance between the fractured bone ends. The centre point of flexion was identified, and its pre-impact angle was compared with that at the time of fracture and at maximal displacement. Contact of the temporary cavity with the fracture was deemed potential contamination, whereas contact with the permanent cavity or retained bullet debris was deemed contaminated. On dissection, periosteal integrity around the fracture site was assessed as well as the fracture pattern.

Four different bullets were used: a 5.56 × 45 mm (NATO SS109), a 9 × 19mm full metal jacket (DM1 1A1B2), a .44 in. semi-jacketed hollow-point (Remington Magnum) and a 7.62 × 39 mm steel core (Factory 71, 1984). Pre- and post-impact bullet velocities were recorded using a Doppler radar and verified with three sky-screen chronographs (MS Instruments, Orpington, UK).

## Results

Of the 42 samples, 22 were shot with a 5.56 × 45 mm, 2 of which were thigh moulds. Twelve were shot with a .44 in., 2 of which were thigh moulds. Four were shot with a 7.62 × 39 mm and four with a 9 × 19 mm; none of these were thigh moulds.

Fracture was produced in 13 of these samples, 5 by the 5.56 × 45 mm and 8 by the .44 in.. No fracture was produced by the 7.62 × 39 mm or 9 × 19 mm. Two of the fractures represented a remote ballistic fracture, both of which were produced by the .44 in. shot 3 cm off the medial cortex of the bone. One was in a rectangular mould and the other in a thigh mould. For the .44 in. bullet, the remaining fractures consisted of two direct fractures and four indirect fractures, all of which were shot with a bullet passage of 2 cm or less from the bone. The five fractures produced by the 5.56 × 45 mm bullet included one direct and four indirect fractures, all shot a maximum of 1 cm off the bone.

**Figure 1:**
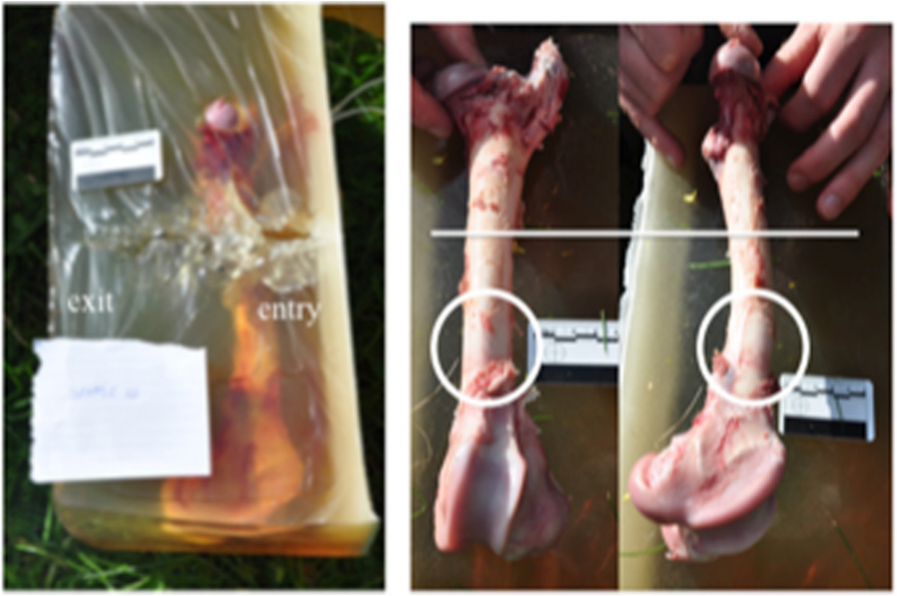
**Representative photographs of the rectangular-shaped gelatine sample, which produced a remote fracture. **On the *left *is the embedded femur with the permanent cavity seen. On the *right *are an anterior and a medial view of the dissected fracture with a *line *representing the bullet height on the bone and a *circle *surrounding the fracture.

**Figure 2:**
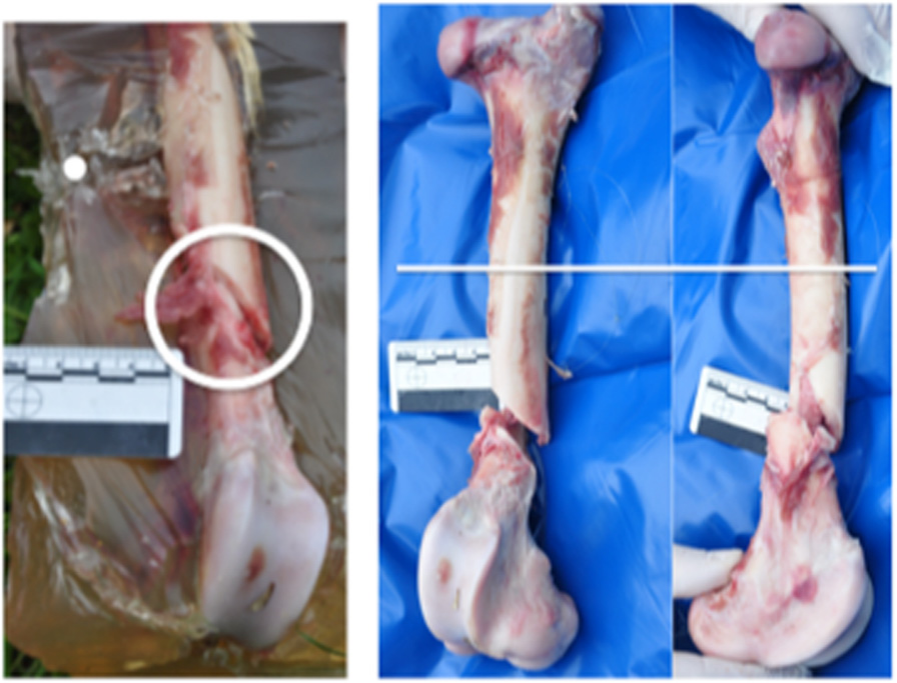
**Representative photographs of the thigh-shaped gelatine sample, which produced a remote fracture. **On the *left *is the embedded femur being dissected with the bullet path shown as a *dot *and the fracture *circled*. On the *right *are an anterior and a medial view of the dissected fracture with a *line *representing the height of the bullet on the bone.

On backlighting of the gel mould, both fractures appeared to have a spiral pattern commencing on the medial cortex 5 cm distal to the bullet tract (junction of middle and distal thirds, 9 cm above the femoral condyles) and continuing to the metaphyseal bone on the lateral cortex (4 cm above the femoral condyles). Gapping of 2 mm was seen on the lateral cortex, but no gapping was identified on the medial cortex (Figures 1 and 2). However, on dissection, an undisplaced and incomplete short oblique fracture line was also identified on both samples. This extended from the gapped lateral cortex to the medial metaphyseal bone, 3 cm above the femoral condyles, ultimately creating a wedge-shaped fracture pattern with its apex being the gapped lateral cortex. A small degree of comminution was seen on the medial cortex of both samples.

**Figure 3:**
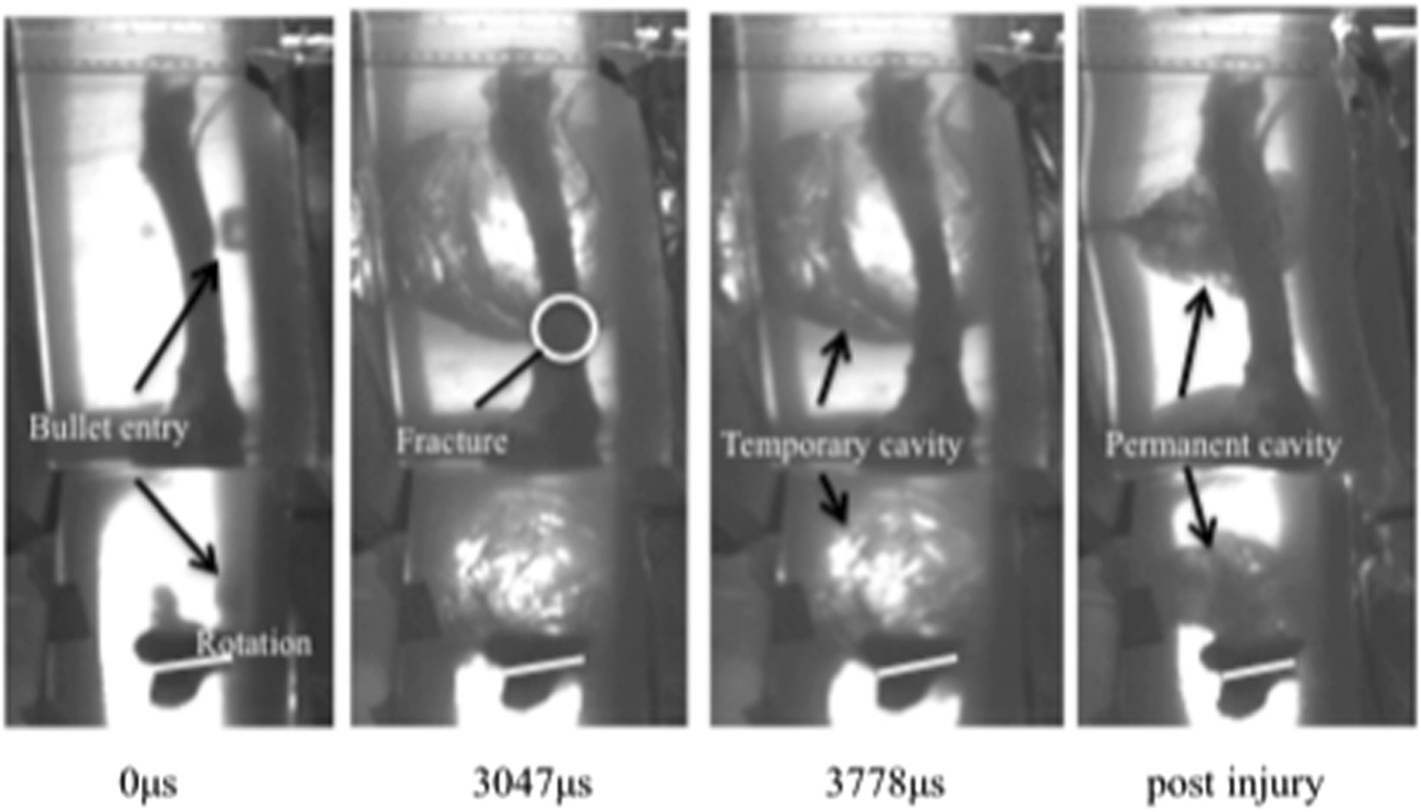
**Time sequence of the remote fracture occurring in a rectangular gelatine block. **From *left *to *right*: pre-impact, time of fracture, time at maximal displacement and then the post-injury residual position.

**Figure 4:**
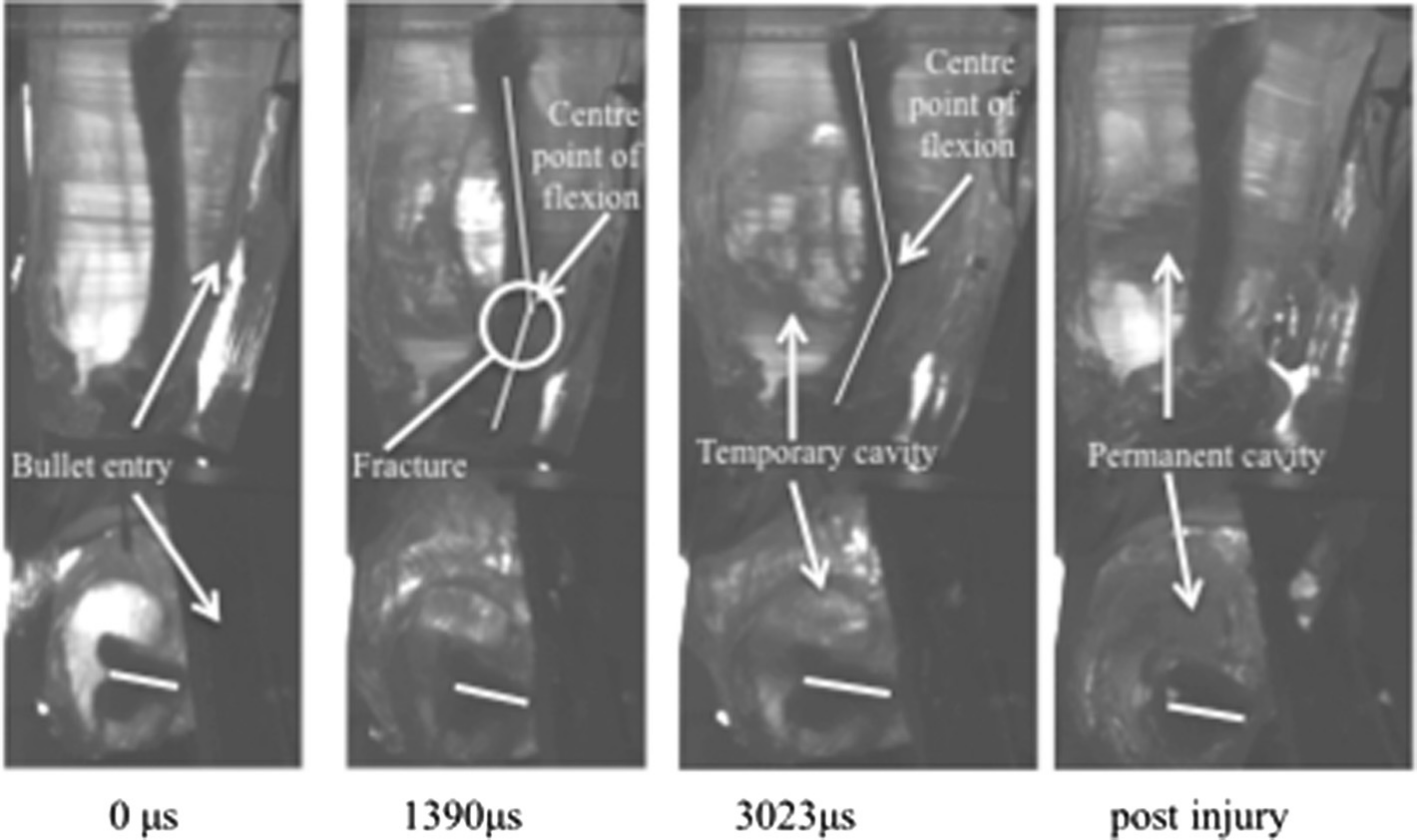
**Time sequence of a remote fracture occurring in a thigh model. **From *left* to *right*: pre-impact, time of fracture, time at maximal displacement and then the post-injury residual position.

On the film, the bone was seen to fracture when the leading edge of the expanding temporary cavity impacts the centre point of flexion (Figures 3 and 4). Both samples were seen to flex 2.5° and displace 3–5 mm laterally before fracturing. No rotation was seen at the time of fracture in either sample, again supporting a wedge-shaped fracture, rather than a spiral one.

Maximal bone flexion was 3° to 4°, with 5.7 to 7.8 mm of fracture displacement but with only 2 mm of bone end separation for either sample. Both fractures were seen to reduce with the collapse of the temporary cavity and only displace 1–2 mm with the temporary cavity's oscillation.

The pre-impact velocity of the .44 in. bullets was 487 m/s (range 480–494 m/s), with energy transfer to the gel, seen in the remote fractures, of 1,723 and 1,477 J for the rectangular and thigh moulds, respectively. This was not significantly different from the other .44 in. gunshot samples (1,477–1,723 J) or the 5.56 × 45mm samples (1,307–1,874 J). However, it was significantly higher than the samples shot with a 9 × 19 mm (207–614 J) or 7.62 × 39 mm (364–511 J) bullet.

The temporary cavity for all .44 in. bullets started at the impact surface of the gel and expanded as a narrow cone encompassing the entire depth of the gel, with its maximal diameter of 16 cm (12–20 cm) occurring at a depth of 15 cm (12–20 cm). This temporary cavity morphology was significantly different to all other bullets tested. For the 5.56 × 45 mm bullets, a narrow cavity (maximum diameter of 4 cm) was followed by an expanding cavity (11–20 cm) at a depth of 14 cm after the bullet had yawed. For the 7.62 × 39 mm and 9 × 19 mm bullets, only a narrow cavity of 10 and 5 cm in diameter, respectively, formed. Note that the values quoted are larger than the pre-impact gelatine mould dimensions because the expanding cavity expands the gelatine and thus temporarily enlarges the gel mould.

In the samples sustaining remote fractures, the temporary cavity was seen to encompass the entire fracture, potentially contaminating the bone ends. However, on dissection, the permanent cavity was not seen to connect with the remote fractures, despite the periosteum having been stripped off the medial cortex of both samples, including the entire base of the wedge fracture. This periosteum had been relayed onto the bone, approximating its original position, by the collapse of the temporary cavity. In addition, the lateral cortex maintained its soft tissue attachments.

## Discussion

Remote ballistic fractures represent a small and highly unique subset of fractures that occur in gunshot trauma. These fractures have been addressed when published as case reports but are often associated with a fall or other injuries, which obscures whether they are actually caused by the gunshot itself [11,12].

Ryan et al. [11] documented six such injuries, out of a total of 143 consecutive femoral fractures due to gunshot injuries presenting to Detroit General Hospital over a 7-year period. Whilst credited for introducing this fracture to the literature, the authors did not further describe or analyze this subset of fractures.

Three years later, Smith and Wheatley [12] presented two more civilian injuries, in which they believed that most notably one patient had sustained such an injury, who had not suffered an associated fall or injury. They described spiral fractures up to 12.5 cm from the bullet tract and proposed that these were unique to the weight-loaded femur. They postulated that they were the result of stress risers acting remotely from the bullet path, with the spiral nature being thought to be due to intrinsic femoral torsion from the eccentric position of the femoral head and the varus bow of the femoral shaft.

However, one problem with this theory originates from the patient history; no patients described a second crack, pop or noise before falling. This suggests that the femur was fractured either at the time of the gunshot or after the fall, but not between.

We propose that this type of fracture is not unique to the weight-bearing limb as none of our samples were loaded. We propose that these fractures, in fact, originate as a direct result of the expansion of the temporary cavity, flexing the femur at its weakest point and developing a tension failure on the opposite cortex to the expanding cavity and a compression wedge expanding towards the near cortex [13,14]. One of the fracture lines of the wedge may remain incomplete and thus imperceptible to plain radiographs, therefore giving the appearance of a spiral fracture on X-ray. The complete fracture line often gapes on its flexed side and may have associated comminution of the compressed side, within the base of the wedge.

Why these fractures occur remotely from the bullet tract is unclear but may be explained in our experimental samples by tension failure occurring in the thinner metaphyseal bone, particularly at its junction with the diaphysis where the bone begins to flare towards the condyles. This region not only represents an area of weakness of the bone, due to widening of the canal, thinning of the cortices and poor bone stock, but also represents an area of angular change, which allows stresses to concentrate [15]. In clinical scenarios not affecting this metaphyseal region, it may be local bone imperfections, abnormalities or vascular permeations that are the weakest points allowing fracture initiation to occur.

We propose that the few steps of mobilization, often reported by patients, are afforded by the residual stability of the undisplaced fracture in compression. In addition, the fight-or-flight response after being shot may dull the pain response allowing mobilization on a fractured limb [16]. However, with the inevitable multidirectional stresses of gait, the fracture displaces, becoming unstable and the patient falls to the ground from mechanical instability and pain.

Exactly why the other calibres and firing distances off the bone failed to produce remote fractures is debatable. The only published series of these injuries involve civilian gunshot wounds, despite anecdotal evidence of their existence in battlefield casualties. Perhaps a large superficial expanding cavity is necessary to distort the bone significantly enough to cause remote fracture and this does not occur with current ‘humane’ military bullets, or perhaps it does occur but has not been reported. Interestingly, we found that if the bullet passed too close to the bone (2 cm or less), an indirect [5] rather than a remote fracture occurs. This phenomenon occurs because the closer the bullet is to the bone, the smaller the temporary cavity's surface area that impacts the bone, and therefore, point loading occurs at the level of the bullet tract causing fracture at this level before the stress waves reach more remote locations. The difference, therefore, between remote ballistic fractures and indirect fractures is that remote fractures occur through the weakest part of the bone, whereas indirect fractures occur directly opposite the bullet tract.

It is uncertain what the infection risk is with potential contamination from the temporary cavity. However, for fracture healing, it is reassuring that the far cortex's periosteal attachments remained intact and that the stripped periosteum was rapidly relayed into its native position with the collapse of the temporary cavity.

This study's low numbers and the variance within organic tissues limit the interpretation of our results. Further research and case reports are therefore needed to fully understand this unique fracture.

## Conclusions

This study presents the first experimentally produced remote ballistic fractures and concludes that these are potentially contaminated, wedge-shaped fractures that are caused by the expansion of the temporary cavity, similar to indirect ballistic fractures, but fracturing through the weakest part of the bone. In addition, despite anecdotal reports in battlefield casualties, this experiment and the current literature only support the existence of this fracture within the civilian setting.

## Competing interests

The authors declare that they have no competing interests.

## Authors’ contributions

DCK was the principal investigator. DJC and IH were integral in the study design, implementation and analysis. SCJL carried out the data analysis and writing, while JCT, MVS and JAK performed the data interpretation and critical revision. All authors read and approved the final manuscript.
